# Epidemiological assessment of the risk of canine mast cell tumours based on the Kiupel two-grade malignancy classification

**DOI:** 10.1186/s13028-018-0424-2

**Published:** 2018-11-03

**Authors:** Anna Śmiech, Brygida Ślaska, Wojciech Łopuszyński, Agnieszka Jasik, Diana Bochyńska, Roman Dąbrowski

**Affiliations:** 1 Sub-Department of Pathomorphology and Forensic Veterinary Medicine, Department and Clinic of Internal Medicine, Faculty of Veterinary Medicine, University of Life Science Lublin, Akademicka 13 St., 20-950 Lublin, Poland; 2 Department of Biological Bases of Animal Production, Faculty of Animal Breeding and Biology, University of Life Science, Akademicka 13 St., 20-950 Lublin, Poland; 3 Department of Pathology, National Veterinary Research Institute, Al. Partyzantów 57 St., 24-100 Pulawy, Poland; 4 Department of Anatomy and Pathology, University Centre of Veterinary Medicine of Jagiellonian University and University of Agriculture, Mickiewicza 21 St., 31-120 Kraków, Poland; 5 Department and Clinic of Animal Reproduction, Faculty of Veterinary Medicine, University of Life Sciences, Akademicka 13 St., 20-950 Lublin, Poland

**Keywords:** Dog, Epidemiological study, Kiupel grade, Mast cell tumours

## Abstract

**Background:**

The degree of differentiation of mast cell tumours (MCTs) is the most important feature and reflects the morphological characteristics and metastatic potential of the tumour and its likely response to treatment and the prognosis. The aim of this study was to epidemiologically analyse the risk of MCT development in dogs according to breed, age, sex, size and anatomical location of the tumour using the Kiupel grading system. The analysis involved 492 dogs selected based on a histopathological assessment of 2763 canine skin tumours. A logistic regression analysis was performed to determine the odds ratios (ORs) with 95% confidence intervals.

**Results:**

Mast cell tumours accounted for 17.8% of all diagnosed canine skin tumours. The highest risk of high-grade MCTs was noted in the Shar-Pei (OR 28.18, P < 0.001) and Weimaraner (OR 6.45, P = 0.023). The highest risk of low-grade MCTs was determined in the Boxer (OR 6.72, P < 0.001), and Pug (OR 6.13, P = 0.027). The scrotum (OR 31.72, P < 0.001), inguinal area (OR 17.69, P < 0.001) and axilla (OR 6.30, P < 0.001) had the highest risk of high-grade MCTs. The risk of high-grade MCTs increased with age and peaked in the oldest dogs, aged 11–16 years (OR 9.55, P < 0.001). A higher risk of low-grade tumours was noted in younger dogs (aged 4–6 years) (OR 8.54, P < 0.001) and females (OR 1.43, P = 0.001). Statistical analysis further revealed a higher risk of both low (OR 3.47, P < 0.001) and high-grade MCTs (OR 1.71, P = 0.006) in medium-sized dogs.

**Conclusions:**

This study demonstrated relationships between Kiupel grading system and phenotypic traits, age and location of canine MCTs confirming the complex biological nature of this tumour.

**Electronic supplementary material:**

The online version of this article (10.1186/s13028-018-0424-2) contains supplementary material, which is available to authorized users.

## Background

Mast cell tumours (MCTs) are characterised by a varied clinical course. They take the form of small, demarcated, single or multiple tumours, they may infiltrate the surrounding tissues and metastasise to lymph nodes and internal organ [1–4]. Although many investigations have focused on identifying the factors determining the probable course of the disease, the degree of histological differentiation is still the most important predictor [5–10] and determines not only the morphological characteristics and metastatic potential of a tumour but also its response to treatment and the prognosis [3, 11, 12]. Before 2011, the most widely used malignancy differentiation system was the three-grade Patnaik scale, which distinguished well, moderately, and poorly differentiated MCTs, referred to as GI, GII, and GIII [13]. Due to the heterogeneous character of moderately differentiated tumours and their unpredictable clinical course, Kiupel et al. [14] proposed a new 2-grade malignancy classification, i.e., low-grade and high-grade, based on the morphology of the cells’ nucleus and the number of mitotic division figures. MCTs with a high malignancy grade are transformations involving at least 7 mitotic division figures, three multinucleated cells, three cells with bizarre nuclei per 10 high-power fields, and 10% of cells with karyomegaly. All other tumours that do not meet these criteria are classified as low-grade (Additional file 1). In accordance with the new classification system, high-grade tumours are characterised by a more aggressive disease course, a tendency to relapse and metastasise, and a shorter patient survival time. The median survival time is approximately 4 months in the case of high-grade MCTs and 2 years for low-grade MCTs [14]. Epidemiological investigations conducted so far have mainly focused on assessing the risk of MCT development in specific dog breeds in a specific geographical region. The risk of this type of tumour in relation to the dog’s age, sex, and body weight and in castrated or sterilised dogs has been demonstrated [15–24]. Only one report has presented an epidemiological analysis of the incidence of MCTs based on the two-grade malignancy [25]. Prognosis is controversial and depends on the location of the MCTs. MCTs can develop in every part of the body, although the most frequent locations include the torso (50–60%), limbs (25–40%), and head and neck (10%) [4]. In turn, MCT locations with a poorer prognosis include the perineal area, perineal–perianal area, and mucocutaneous junctions [1, 2, 4, 12]. Some authors claim that a worse prognosis is often associated with the development of poorly differentiated MCTs, whereas other researchers relate it to difficulties in performing surgery [26, 27]. Therefore, a retrospective analysis of the risk of development of low- and high-grade MCTs could be extremely helpful for prognosis. There are no epidemiological studies in the veterinary literature based on the Kiupel two-grade classification of malignancy, which is the basic prognostic factor determining the course of the disease.

The aim of this study was to conduct an epidemiological analysis of the risk of MCT development in dogs in relation to other skin tumours and to use the data in the prognosis of the neoplastic disease. Relationships between the dog’s breed, age, sex, size, anatomical location of the tumour, and degree of MCT malignancy specified by the Kiupel two-grade malignancy scale were assessed.

## Methods

The analysis involved 492 dogs of 77 breeds and crossbreed dogs diagnosed with skin MCTs, which were selected based on a histopathological assessment of 2763 canine skin tumour cases diagnosed at the Sub-Department of Pathomorphology and Forensic Veterinary Medicine, University of Life Sciences in Lublin, Poland from 2003 to 2016. Due to the small number of dogs, 51 breeds represented by 31 individuals with MCTs and 433 dogs with skin tumours were classified into one group, i.e., other breeds. The analysis was conducted in 26 breeds, crossbreed dogs, and the group of other breeds. The tumour samples for histopathological examination originated from dogs treated with surgical resection of the skin tumour, which was performed at the Veterinary Clinic, University of Life Sciences in Lublin and at private veterinary clinics in Poland. Slides for microscopic evaluation were routinely stained with haematoxylin and eosin as well as toluidine blue. The histopathological analysis of the MCTs was performed according to the WHO classification based on the two-grade malignancy scale of Kiupel et al. [14, 28]. Tumours sampled before 2011 and evaluated according to the Patnaik scale were reclassified by three pathologists. Clinical data on the dogs’ breed, age, sex, and tumour location were derived from records delivered to the Department, together with tissue submitted for histopathological examination. Only dogs with a complete set of data were qualified for inclusion in the study; hence, 78 cases were excluded from the analyses. The analyses were performed on tumours diagnosed in an individual for the first time.

Dogs were divided into three groups according to size: small (S), estimated wither height (EWH) 30–45 cm, medium (M, EWH 45–60 cm), and large (L, EWH > 60 cm) [29]. The crossbreed dogs were excluded from the body-size assessment. Additionally, four age groups were distinguished: (1) dogs aged 0–3 years, (2) 4–6 years, (3) 7–10 years, and (4) 11–16 years. Eleven tumour locations were distinguished: (1) head, (2) neck, (3) torso, (4) thoracic limb, (5) axilla, (6) pelvic limb, (7) inguinal area, (8) perineum (9) scrotum, (10) anus, and (11) tail.

The risk of MCT development according to breed, sex, size, location, and age range was determined based on the odds ratio (OR). The control (reference) group comprised dogs with skin tumours diagnosed at the Sub-Department of Pathomorphology and Forensic Veterinary Medicine, University of Life Sciences in Lublin during the same period, i.e., from 2003 to 2016. A logistic regression analysis was performed to determine the ORs with 95% confidence intervals (CIs). For dogs assigned to a breed, ORs were calculated by comparing the MCT incidence in the analysed breed with that in the other breeds diagnosed with skin tumours (control group). Analogous calculations were conducted for tumour location. For the calculations of ORs relative to age, the dogs were divided into four age groups, and the younger animals (up to 3 years of age) were regarded as the basal group. Small dogs and males were the basal groups in the determination of ORs for size and sex, respectively. The analysis was conducted using the Statistica 9.1 program (StatSoft^®^, Cracow Poland). Values of P < 0.05 were considered significant.

## Results

The 492 cases of MCTs accounted for 17.8% of all skin tumours. Among the skin tumours, 19.6% sebaceous and sweat gland tumours, 15.9% histiocytic tumours, 10.9% epidermal tumours, 10.8% follicular tumours and 5.0% melanocytic tumours were identified. In turn, mesenchymal neoplasms and other tumours accounted for 20.0% of the examined skin tumours. According to the two-grade classification of Kiupel et al. [14], low-grade tumours were dominant, representing 75.8%; the other cases were classified as high-grade tumours (Table 1). The greatest proportion of MCTs was detected in the Boxer breed (19.1%), of which 96.8% were classified as low grade. Furthermore, a high percentage of MCTs were noted in Labrador Retrievers, American Staffordshire Terriers, Golden Retrievers, French Bulldogs, Dachshunds, and Shar-Peis (ranging from 2.6 to 9.9%) (Table 1). In terms of location, the greatest numbers of MCTs were noted on the torso (36.9%); they were dominated by low-grade tumours (80.2%). In turn, the highest frequency of high-grade tumours (73.3%) was noted in the inguinal region (Table 2). Data presenting the frequency of MCTs in relation to the dog’s sex, size, and the four age groups in the analysed dog population are shown in Table 3. The highest risk of MCT development, compared with that of other skin tumours, was detected in five breeds: Shar-Pei, Boxer, American Staffordshire Terrier, Labrador Retriever, and French Bulldog. In turn, the lowest incidence was found in Cocker Spaniel, German Shepherd, and Yorkshire Terrier (Table 4). The statistical analysis based on the Kiupel two-grade malignancy scale revealed the highest risk of high-grade MCT development in three breeds: Shar-Pei, Weimaraner and American Staffordshire Terrier (Table 4). The highest risk of low-grade MCTs was observed in five breeds: Boxer, Pug, Labrador Retriever, American Staffordshire Terrier and French Bulldog (Table 4).

**Table 1:** Frequency of MCTs in various breeds of dogs according to the Kiupel grading system

Breed	All MCTs		Kiupel grade				Control group^d^	
			Low		High			
	N	%	N	%^a^	N	%^b^	N	%
Boxer	94	19.1	91	96.8	3	3.1	104	4.5
Labrador	49	9.9	40	81.6	9	18.3	96	4.2
American Staffordshire Terrier	30	6.1	22	73.3	8	26.6	53	2.3
Golden Retriever	19	3.8	16	84.2	3	15.7	64	2.8
French Bulldog	15	3.0	11	73.3	4	26.6	34	1.5
Dachshund	14	2.8	5	35.7	9	64.2	93	4.0
Shar-Pei	13	2.6	1	7.6	12	92.3	9	0.4
Bernese Mountain Dog	11	2.2	7	63.6	4	36.3	39	1.7
German Shepherd	9	1.8	6	66.6	3	33.3	211	9.2
Miniature Schnauzer	7	1.4	6	85.7	1	14.2	52	2.2
Irish Setter	5	1.0	5	100.0	0	0.0	23	1.0
Standard Schnauzer	5	1.0	3	60.0	2	40.0	29	1.2
Cocker Spaniel	4	0.8	4	100.0	0	0.0	91	4.0
Doberman	4	0.8	3	75.0	1	25.0	40	1.7
Maltese	4	0.8	4	100.0	0	0.0	9	0.4
Bull Terrier	3	0.6	3	100.0	0	0.0	15	0.6
Pug	3	0.6	3	100.0	0	0.0	3	0.1
Polish Tatra Sheepdog	3	0.6	2	66.6	1	33.3	7	0.3
Siberian Husky	3	0.6	3	100.0	0	0.0	33	1.4
Weimaraner	3	0.6	1	33.3	2	66.6	6	0.2
Saint Bernard	2	0.4	2	100.0	0	0.0	14	0.6
Jack Russell Terrier	2	0.4	1	50.0	1	50.0	7	0.3
Caucasian Shepherd	2	0.4	2	100.0	0	0.0	7	0.3
Miniature Poodle	2	0.4	1	50.0	1	50.0	4	0.1
Standard Poodle	2	0.4	2	100.0	0	0.0	22	0.9
Yorkshire Terrier	2	0.4	2	100.0	0	0.0	81	3.5
Other breeds	31	6.3	24	77.4	7	22.5	433	19.0
Crossbreed	151	30.6	103	68.2	48	31.7	692	30.4
Total	492	17.8^c^	373	75.8	119	24.1	2271	82.2

^a^Percentage of dogs with low-grade MCTs within a given breed of dog with MCTs

^b^Percentage of dogs with high-grade MCTs within a given breed of dog with MCTs

^c^Percentage of dogs with MCTs among all tested dogs

^d^Total number of dogs with other skin tumours within a given breed

**Table 2:** Frequency of MCT grades by tumour location

Location	All MCTs		Kiupel grade				Control group^c^	
			Low		High			
	N	%	N	%^a^	N	%^b^	N	%
Torso	182	36.9	146	80.2	36	19.7	620	27.3
Pelvic limb	93	18.9	80	86.0	13	13.9	331	14.5
Head	55	11.1	45	81.8	10	18.1	506	22.2
Thoracic limb	55	11.1	47	85.4	8	14.5	298	13.1
Axilla	22	4.4	8	36.3	14	63.6	47	2.0
Neck	21	4.2	16	76.1	5	23.8	109	4.8
Scrotum	21	4.2	9	42.8	12	57.1	8	0.3
Inguinal area	15	3.0	4	26.6	11	73.3	13	0.5
Perineum	12	2.4	5	41.6	7	58.3	30	1.3
Tail	10	2.0	10	100.0	0	0.0	79	3.4
Anus	6	1.2	3	50.0	3	50.0	230	10.1
Total	492	100.0	373	75.8	119	24.1	2271	100.0

^a^Percentage of low-grade MCTs in a given location

^b^Percentage of high-grade MCTs in a given location

^c^Total number of dogs with other skin tumours in a given location

**Table 3:** Frequency of MCT grade by age, size and sex

Variable	All MCTs	Kiupel grade		Control group^b^
		Low	High	
Age (years)				
0–3	15	11	4	398
	3.0%	2.9%	3.3%	17.5%
4–6	127	110	17	466
	25.8%	29.4%	14.2%	20.5%
7–10	262	210	52	928
	53.2%	56.3%	43.7%	40.8%
11–16	88	42	46	479
	17.8%	11.2%	38.6%	21.0%
M ± SD	8.09 ± 2.72	7.64 ± 2.42	9.52 ± 3.10	7.44 ± 3.73
Size^a^				
Small	65	46	19	474
	19.0%	17.0%	26.7%	30.0%
Medium	207	172	35	510
	60.7%	63.7%	49.3%	32.3%
Large	69	52	17	594
	20.2%	19.2%	23.9%	37.6%
Sex				
Male	274	205	69	1444
	55.6%	54.9%	57.9%	63.5%
Female	218	168	50	827
	44.3%	45.0%	42.0%	36.4%
Total	492	373	119	2271

*M* mean, *SD* standard deviation

^a^The analysis did not take into account crossbreed dogs, whose size was not recognized

^b^Total number of dogs with other skin tumours of a given age, size and sex

**Table 4:** Odds ratios (ORs) and 95% confidence intervals (CIs) for particular grades of MCTs in various breeds of dogs

Breed	All MCTs		Kiupel grade			
			Low		High	
	OR (95% CI)	P	OR (95% CI)	P	OR (95% CI)	P
American Staffordshire Terrier	2.71 (1.71–4.3)	< 0.001	2.62 (1.57–4.36)	< 0.001	3.01 (1.40–6.49)	0.005
Bernese Mountain Dog	1.30 (0.66–2.57)	0.435	1.09 (0.48–2.46)	0.827	1.99 (0.69–5.66)	0.197
Boxer	4.92 (3.65–6.63)	< 0.001	6.72 (4.94–9.14)	< 0.001	0.53 (0.16–1.72)	0.297
Bull Terrier	0.92 (0.26–3.19)	0.899	1.21 (0.35–4.23)	0.755	–	–
Caucasian Shepherd	1.32 (0.27–6.37)	0.73	1.74 (0.36–8.42)	0.489	–	–
Cocker Spaniel	0.19 (0.07–0.53)	0.002	0.26 (0.09–0.71)	0.009	–	–
Dachshund	0.68 (0.38–1.21)	< 0.001	0.16 (0.07–0.36)	< 0.001	0.25 (0.08–0.80)	0.019
Doberman	0.45 (0.16–1.28)	0.137	0.45 (0.13–1.46)	0.187	0.47 (0.06–3.46)	0.461
French Bulldog	2.06 (1.11–3.82)	0.021	1.99 (1.00–3.98)	0.049	2.28 (0.79–6.55)	0.123
German Shepherd	0.18 (0.09–0.35)	< 0.001	0.16 (0.07–0.36)	< 0.001	0.25 (0.08–0.80)	0.019
Golden Retriever	1.38 (0.82–2.33)	0.221	1.54 (0.88–2.70)	0.127	0.89 (0.27–2.88)	0.848
Irish Setter	1.00 (0.38–2.65)	0.994	1.32 (0.50–3.51)	0.568	–	–
Jack Russell Terrier	1.32 (0.27–6.37)	0.73	0.86 (0.10–7.08)	0.896	2.74 (0.33–22.46)	0.347
Labrador	2.50 (1.75–3.58)	< 0.001	2.72 (1.84–4.00)	< 0.001	1.85 (0.91–3.76)	0.088
Maltese	2.06 (0.63–6.71)	0.231	2.72 (0.83–8.89)	0.097	–	–
Miniature Poodle	2.31 (0.42–12.66)	0.334	1.52 (0.17–13.66)	0.707	4.80 (0.53–43.30)	0.162
Miniature Schnauzer	0.61 (0.27–1.36)	0.232	0.69 (0.29–1.63)	0.408	0.36 (0.05–2.63)	0.316
Polish Tatra Sheepdog	1.98 (0.51–7.70)	0.322	1.74 (0.36–8.42)	0.489	2.74 (0.33–22.46)	0.347
Pug	4.63 (0.93–23.04)	0.061	6.13 (1.23–30.48)	0.027	–	–
Saint Bernard	0.65 (0.14–2.90)	0.581	0.86 (0.19–3.83)	0.853	–	–
Shar-Pei	6.82 (2.89–16.04)	< 0.001	0.67 (0.08–5.34)	0.71	28.18 (11.62–68.34)	< 0.001
Siberian Husky	0.41 (0.12–1.36)	0.147	0.55 (0.16–1.80)	0.323	–	–
Standard Poodle	0.41 (0.09–1.78)	0.238	0.55 (0.12–2.35)	0.421	–	–
Standard Schnauzer	0.79 (0.30–2.06)	0.635	0.62 (0.19–2.06)	0.443	1.32 (0.31–5.60)	0.705
Weimaraner	2.31 (0.57–9.29)	0.236	1.01 (0.12–8.45)	0.989	6.45 (1.28–32.31)	0.023
Yorkshire Terrier	0.11 (0.02–0.45)	0.002	0.14 (0.03–0.59)	0.007	–	–

**Fig. 1:**
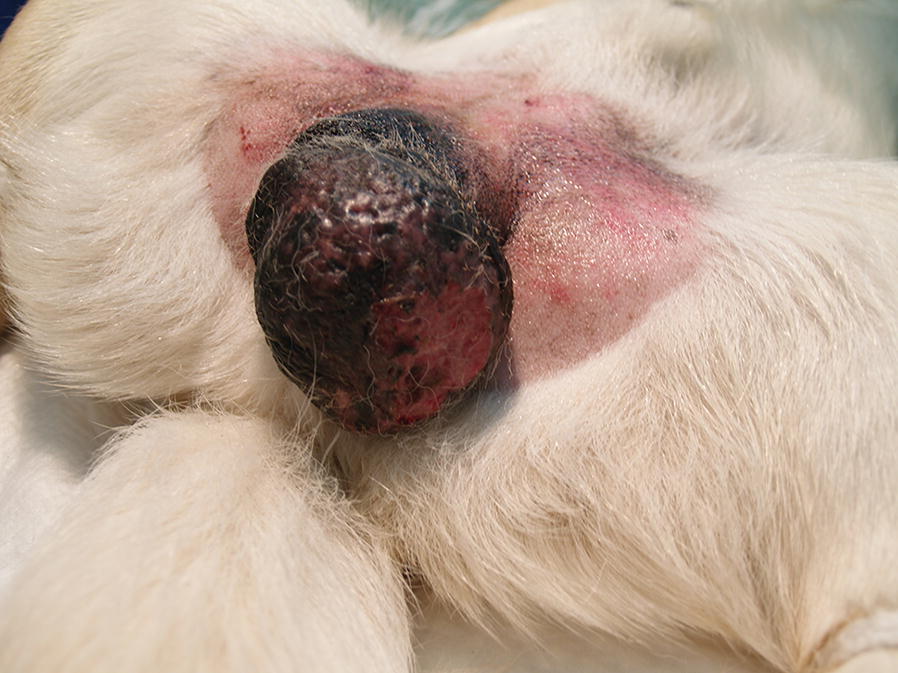
High-grade MCT in the scrotum of a 9-year-old Labrador

The scrotum was characterised by the highest risk of MCT development of all the skin tumour locations (Table 5). This region exhibited a substantially greater risk of high-grade tumour development (Fig. 1), although the OR indicator for low-grade MCTs was also high (Table 5). Other localities with high OR values included the inguinal area, axilla and torso (Table 5). Higher OR values were determined for high-grade tumours in the inguinal area and axilla. In turn, the torso was found to be the region with the highest risk of low-grade tumour development (Table 5).

**Table 5:** Odds ratios (ORs) and 95% confidence intervals (CIs) for particular grades of MCTs by tumour location

Location	All MCTs		Kiupel grade			
			Low		High	
	OR (95% CI)	P	OR (95% CI)	P	OR (95% CI)	P
Anus	0.11 (0.04–0.24)	< 0.001	6.99 (2.68–18.24)	< 0.001	31.72 (12.70–79.23)	< 0.001
Axilla	2.21 (1.32–3.71)	0.003	1.03 (0.48–2.21)	0.925	6.30 (3.36–11.82)	< 0.001
Head	0.44 (0.32–0.59)	< 0.001	0.48 (0.34–0.67)	< 0.001	0.32 (0.16–0.62)	0.001
Inguinal area	5.46 (2.58–11.55)	< 0.001	1.88 (0.61–5.80)	0.271	17.69 (7.74–40.40)	< 0.001
Neck	0.88 (0.54–1.42)	0.614	0.88 (0.52–1.52)	0.667	0.87 (0.34–2.17)	0.766
Pelvic limb	0.72 (0.56–0.94)	0.015	0.62 (0.47–0.81)	0.001	1.38 (0.77–2.49)	0.276
Pelvic limb	0.72 (0.56–0.94)	0.015	0.62 (0.47–0.81)	0.001	1.38 (0.77–2.49)	0.276
Perineum	1.93 (0.97–3.81)	0.058	1.05 (0.40–2.73)	0.92	4.83 (2.07–11.27)	< 0.001
Scrotum	12.61 (5.55–28.64)	< 0.001	6.99 (2.68–18.24)	< 0.001	31.72 (12.70–79.23)	< 0.001
Tail	0.58 (0.3–1.13)	0.112	0.77 (0.39–1.51)	0.453	–	–
Thoracic limb	0.84 (0.62–1.14)	0.274	0.96 (0.69–1.34)	0.836	0.48 (0.23–1.00)	0.05
Torso	1.59 (1.29–1.95)	< 0.001	1.74 (1.38–2.18)	< 0.001	1.17 (0.78–1.75)	0.43

A higher risk of MCT development was noted in females than in males, including a higher risk of low-grade MCTs (Table 6). There was an increased risk of MCT development in older dogs aged 4–6 years and 7–10 years compared with that in the youngest dog group (less than 3 years old) (Table 6). Similar correlations were observed for high-grade tumours, but the highest OR value was noted in the oldest dog group (aged 11–16 years). The highest risk of low-grade tumours was reported for younger dogs aged 4–6 years compared with the youngest group (less than 3 years old) (Table 6). The statistical analysis based on size revealed a higher risk of low- and high-grade MCTs in the medium-sized breeds than in the small ones, whereas no significant correlations were found for the large breeds (Table 6).

**Table 6:** Odds ratios (ORs) and 95% confidence intervals (CIs) for particular grades of MCTs by age, size and sex

Variable	All MCTs		Kiupel grade			
			Low		High	
	OR (95% CI)	P	OR (95% CI)	P	OR (95% CI)	P
Sex						
Male	1 (base)	–	1 (base)	–	1 (base)	–
Female	1.38 (1.14–1.69)	0.001	1.43 (1.14–1.78)	0.001	1.26 (0.87–1.83)	0.217
Age (years)						
0–3	1 (base)	–	1 (base)	–	1 (base)	–
4–6	7.23 (4.16–12.55)	< 0.001	8.54 (4.53–16.10)	< 0.001	3.63 (1.21–10.87)	0.021
7–10	7.49 (4.39–12.77)	< 0.001	8.18 (4.41–15.18)	< 0.001	5.57 (2.00–15.52)	0.001
11–16	4.87 (2.77–8.56)	< 0.001	3.17 (1.61–6.24)	0.001	9.55 (3.41–26.77)	< 0.001
Size						
Small	1 (base)	–	1 (base)	–	1 (base)	–
Medium	2.96 (2.18–4.01)	< 0.001	3.47 (2.45–4.92)	< 0.001	1.71 (0.96–3.03)	0.066
Large	0.84 (0.59–1.21)	0.366	0.90 (0.59–1.36)	0.626	0.71 (0.36–1.38)	0.321

## Discussion

Mast cell tumours accounted for 17.8% of all the examined skin tumours which corresponds to the frequency found in other studies (7–21%) [4, 21, 30]. The results of this study indicated an increased risk of MCT development in five breeds: Shar-Pei, American Staffordshire Terrier, Labrador Retriever, French Bulldog, and Boxer (Table 4). The results most similar to those found in our study were obtained in investigations conducted in the UK, where the highest risk was predicted for Boxers, Labrador Retrievers, Golden Retrievers, and Staffordshire Bull Terriers [22]. Other studies conducted in the UK demonstrated an increased risk of MCT development in Weimaraners as well [20]. Our study confirmed the increased rate of high-grade MCT development in this breed. Epidemiological studies conducted in the USA showed that breeds such as Boxers, Vizslas, Rhodesian Ridgebacks, Boston Terriers, Weimaraners, and Chinese Shar-Peis were more susceptible to MCTs [21]. Differences in MCT incidence among different breeds is associated with the geographical area and the selection of the control population, which in some investigations comprised insured populations [18, 22], dogs registered with kennel associations (Kennel Club registrations) [19, 22], or hospitalised dogs [21, 22]. The control group in our study comprised dogs with skin tumours. Regardless of the geographic area and the control population, all epidemiological studies have revealed an increased risk of MCT development in Boxers [19–23]. The present study confirmed these observations. Moreover, the statistical analysis revealed an increased incidence of MCTs in American Staffordshire Terriers and French Bulldogs. There is a hypothesis that Boxers, American Staffordshire Terriers and French Bulldogs may be related and have a common ancestor in their phylogenetic development [31]. The present study demonstrated a high risk of MCTs in the Shar-Pei simultaneously with an increased risk of high-grade MCTs (Table 4). Our results confirm previous reports of greater susceptibility of this breed to MCTs characterised by a higher malignancy grade and a worse clinical course [32–34]. As shown previously, the Labrador Retriever breed is at increased risk of this type of tumour [22, 23]. This finding was also confirmed in the present study, which additionally revealed a higher risk of low-grade MCTs (Table 3). Recent investigations suggest that low levels of 25(OH)D3 might be a risk factor for MCTs in this breed [35]. Available published data show that Boxers and Pugs are characterised by higher susceptibility to low-grade MCTs [25, 32, 34, 36, 37]. Our epidemiological analysis confirmed these observations and demonstrated an increased risk of low-grade MCTs in French Bulldog and American Staffordshire Terrier. In the latter breed, a higher risk of high-grade MCTs was revealed as well (Table 3), which may be related to the phylogenetic origin. The American Staffordshire Terrier is a cross between the Bulldog and the Terrier. In Bulldogs, a higher risk of low-grade MTC occurrence has been observed [25]. Although there are no reports on the occurrence of high-grade MCT in Terriers, genetic factors should be considered. Other mechanisms in addition to genetic factors probably play an important role and may be responsible for the biological behaviour of tumours in a given breed. Investigations of mitochondrial DNA conducted in recent years have demonstrated somatic mutations in the mitochondrial DNA D-loop in MCTs, which may also be associated with neoplastic transformation [38]. The present epidemiological analysis also showed a reduced risk of MCT development in three breeds, i.e., German Shepherd, Yorkshire Terrier, and Cocker Spaniel (Table 3), which is consistent with previous studies [20–22].

The veterinary literature contains many discrepancies regarding the risk of development of MCTs in females and males. Most reports confirm the absence of a correlation between the animal’s sex and MCT development [20, 33]. The present results showed a higher risk of MCT development in females (OR 1.38, P = 0.001), with a concurrent tendency towards low-grade tumours (OR 1.43, P = 0.001) (Table 5). A study conducted by Mochizuki et al. [25] reported a greater number of high-grade tumours in males and non-castrated dogs. In turn, some published data suggest that castration and sterilisation increase the MCT risk [23, 24, 39]. These data imply that the role of sex hormones in MCT development is not fully understood, and further investigations are required to elucidate this issue.

The present study showed correlations between the anatomical location of the tumour and the presence of MCTs. The statistical analysis demonstrated that the scrotum had the greatest risk of MCT development of all the skin tumour locations. The results also indicated that this area was susceptible to a substantially higher risk of high-grade tumours (OR 31.72, P < 0.001), although the OR value for low-grade MCTs was also high (OR 12.61, P < 0.001) (Table 4). Other regions predisposed to the development of MCTs were the inguinal area and axilla (Table 4). As shown in the literature, the inguinal, scrotal, and perianal areas, as well as the mucocutaneous junctions, are tumour locations characterised by a worse prognosis [1, 2, 4, 12, 27]. However, it should be borne in mind that the worse prognosis may be associated with the difficulty of applying an appropriate surgical procedure and incomplete tumour resection [1, 3]. The present results confirmed the tendency towards the development of high-grade MCTs in the inguinal and axillary regions (Table 4). As reported by Govier [26], mechanical irritation and chronic inflammation may contribute to the development of this tumour. The inguinal and axillary regions are exposed to mechanical irritation, which may contribute to the worse course of the disease. The statistical analysis revealed an increased risk of the development of MCTs on the torso and confirmed this region’s predilection for the occurrence of low-grade MCTs (Table 4).

Mast cell tumours can develop in dogs at all ages, but most cases are diagnosed between 7.5 and 9 years of age [2, 4, 12, 40]. The present study confirmed a higher MCT risk in older dogs aged 4–6 (OR 7.23, P < 0.001) and 7–10 years (OR 7.49, P < 0.001) than in the younger group (Table 5). Shoop et al. [20] found a 41-fold higher risk of MCT development in 10-year-old dogs compared with that in dogs aged 2 years. In turn, Villamil et al. [21] observed an increased MCT incidence in dogs older than 7 years. The statistical analyses presented in this study revealed interesting correlations between a dog’s age and the malignancy grade of MCTs. The comparison with the youngest dogs revealed that the risk of high-grade MCT development increased with age, reaching a maximum value in the oldest group of dogs, aged 11–16 years. In the case of low-grade tumours, the risk was higher in younger dogs, aged 4–6, and declined in the oldest group, aged over 11 years (Table 5). In previous epidemiological studies, no correlations were demonstrated between age and the risk of various-grade MCTs. An investigation conducted by Mochizuki et al. [25] showed an increased risk of mast cell malignancies, mainly in non-castrated males. The results of the present statistical analyses based on the dog’s size revealed a higher risk of low- and high-grade MCTs in medium-sized breeds than in small breeds. No such correlation was found for large breeds (Table 5). In contrast, White et al. [23] reported slightly different results, i.e., a several-fold higher risk of MCT development in large and giant breeds than in small ones; however, that analysis was based on body weight rather than height at the withers.

## Conclusions

This study demonstrated relationships between Kiupel grading system and phenotypic traits, age and location of canine MCTs confirming the complex biological nature of this tumour. Retrospective studies conducted in large animal populations present a valuable contribution to knowledge about the clinical nature of MCTs. Data obtained in the present study can be used for the prediction of to determine the impact of various risk factors in breeds that are predisposed to the development of MCTs.

## Additional file

**Additional file 1.** Photomicrographs of MCT. *a*: Microscopic Image of High-Grade MCT. Note mitotic figure (*arrow*) and karyomegaly (*arrow head)*. Haematoxylin and eosin. *b*: Microscopic Image of Low-Grade MCT. Note round to ovoid nuclei with scattered chromatin. Haematoxylin and eosin.

## Abbreviations

Cl: confidence interval
MCTs: mast cell tumours
OR: odds ratio
