# Supplementary material for: Epidemiological assessment of the risk of canine mast cell tumours based on the Kiupel two-grade malignancy classification
Source: Acta Vet Scand. 2018 Nov 3;60:70. doi: 10.1186/s13028-018-0424-2 (PMC6215678; doi:10.1186/s13028-018-0424-2)

**Additional file 1.**

**Photomicrographs of MCT**.

*a*: Microscopic Image of High-Grade MCT. Note mitotic figure (*arrow*) and karyomegaly (*arrow haed* ). Haematoxylin and eosin. Bar = 20μm

*b*: Microscopic Image of Low-Grade MCT. Note round to ovoid nuclei with scattered chromatin. Haematoxylin and eosin. Bar = 20μm

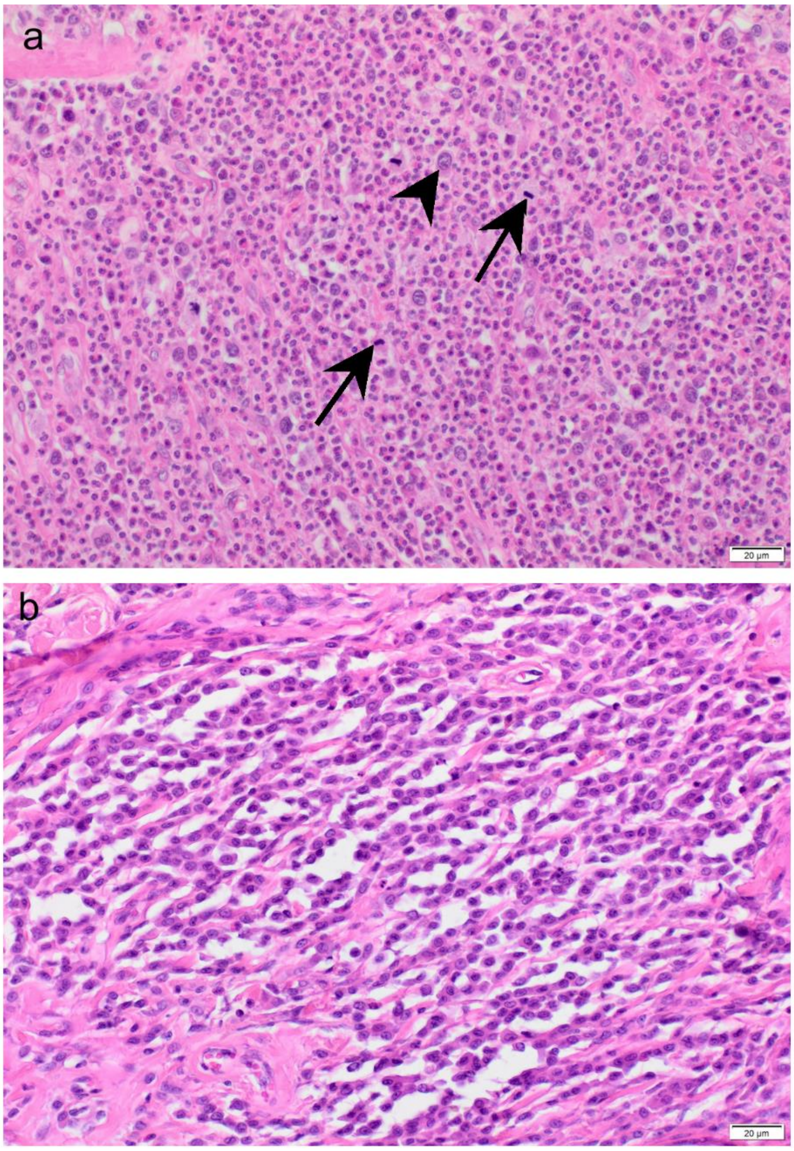

Supplement: Supplementary file 1 — Additional file 1. Photomicrographs of MCT. a: Microscopic Image of High-Grade MCT. Note mitotic figure (arrow) and karyomegaly (arrow head). Haematoxylin and eosin. b: Microscopic Image of Low-Grade MCT. Note round to ovoid nuclei with scattered chromatin. Haematoxylin and eosin. [file 13028_2018_424_MOESM1_ESM.pdf]
